# Rheumatoid arthritis and the incidence of influenza and influenza-related complications: a retrospective cohort study

**DOI:** 10.1186/1471-2474-13-158

**Published:** 2012-08-27

**Authors:** William A Blumentals, Anna Arreglado, Pavel Napalkov, Stephen Toovey

**Affiliations:** 1 Hoffmann-La Roche, Inc, Nutley, NJ, USA; 2 Genentech, Inc, South San Francisco, CA, USA; 3 Royal Free and University College Medical School, London, UK; 4 Burggartenstrasse 32, 4103, Bottmingen, Switzerland

## Abstract

**Background:**

Patients with rheumatoid arthritis (RA) are known to be at increased risk of infection, particularly if they are taking drugs with immunomodulatory effects. There is a need for more information on the risk of influenza in patients with RA.

**Methods:**

A retrospective cohort study was carried out using data gathered from a large US commercial health insurance database (Thomson Reuters Medstat MarketScan) from 1 January 2000 to 31 December 2007. Patients were ≥18 years of age, with at least two RA claims diagnoses. The database was scanned for incidence of seasonal influenza and its complications on or up to 30 days after an influenza diagnosis in RA patients and matched controls. Other factors accounted for included medical conditions, use of disease-modifying anti-rheumatic drugs (DMARDs), use of biological agents, influenza vaccination and high- or low-dose corticosteroids. Incidence rate ratios (IRRs) were calculated for influenza and its complications in patients with RA.

**Results:**

46,030 patients with RA and a matching number of controls had a median age of 57 years. The incidence of influenza was higher in RA patients than in controls (409.33 vs 306.12 cases per 100,000 patient-years), and there was a 2.75-fold increase in incidence of complications in RA. Presence or absence of DMARDs or biologics had no significant effect. The adjusted IRR of influenza was statistically significant in patients aged 60–69 years, and especially among men. A significantly increased rate of influenza complications was observed in women and in both genders combined (but not in men only) when all age groups were combined. In general, the risk of influenza complications was similar in RA patients not receiving DMARDs or biologics to that in all RA patients. Pneumonia rates were significantly higher in women with RA. Rates of stroke/myocardial infarction (MI) were higher in men, although statistical significance was borderline.

**Conclusions:**

RA is associated with increased incidence of seasonal influenza and its complications. Gender- and age-specific subgroup data indicate that women generally have a greater rate of complications than men, but that men primarily have an increased rate of stroke and MI complications. Concomitant DMARD or biological use appears not to significantly affect the rate of influenza or its complications.

## Background

Patients with autoimmune rheumatic diseases have a risk of infection approximately double that of age- and gender-matched controls [1,2]. The literature suggests that, even before the use of corticosteroids became widespread, patients with rheumatoid arthritis (RA) had an increased susceptibility to infection [3]. This is supported by the observation that up to 40% of patients with septic arthritis have RA [4].

Notable diseases in patients with RA include pulmonary infections, particularly pneumonia [5]. The increased risk of infection in patients with chronic rheumatic or autoimmune disease is reported to be linked to compromised immunological functioning and to the immunosuppressive therapies used to control these disorders and their organ-specific complications [2]. Over the past decade, there has been increasing interest in reports of serious infections in patients receiving biological therapies for diseases that include RA [6-8].

A large case-control study based on data from 443 UK general practices, clarified a number of conditions known to increase the risk of community-acquired pneumonia (CAP), and identified seven new independent risk factors [9]. Conditions increasing risk included RA. Among 17,172 incident cases of all ages diagnosed with CAP between 1995 and 2005, the adjusted odds ratio (OR) for pneumonia associated with at least one of the already established risk factors [10] was 2.29 (95% confidence interval [CI]: 2.20, 2.39). After adjustment for known risk factors and confounders, the OR for risk of pneumonia in 387 patients with RA was 1.84 (95% CI: 1.62, 2.10).

Although the Mayo Clinic, for example, lists chronic illness and a weakened immune system as risk factors for influenza and its complications [11], little is known of the risk of influenza specifically in patients with RA. We have carried out a retrospective cohort study based on data gathered over 8 years from a large proprietary US healthcare database, in order to clarify further the effect of RA on the incidence of seasonal influenza and its related complications in adults.

## Methods

### Study design

This was a retrospective cohort study in patients included in the Thomson Reuters Medstat MarketScan Database from 1 January 2000 to 31 December 2007 [12]. Note that data collection preceded the appearance of the 2009 pandemic strain of influenza A (H1N1). Medstat MarketScan (http://marketscan.thomsonreuters.com/marketscanportal/) consists of three core commercial health insurance databases (Commercial, Medicare Supplemental and Medicaid) that cover more than 130 million patients recorded since 1995. The databases link outpatient prescription drug data with inpatient and outpatient claims files via unique encrypted patient identifiers, and are used extensively by health and outcomes researchers for clinical decision support and healthcare resource allocation planning, and to study epidemiological patterns. In compliance with the Health Insurance Portability and Accountability Act (HIPAA), all patient data used in this study were de-identified. The project was therefore exempt from Institutional Review Board approval.

### Study population and outcomes

Patients were ≥18 years of age, with at least two RA claims diagnoses (ICD-9 codes 714.0 or 714.2) [13] on separate occasions from 1 January 2000 to 31 December 2007. Influenza cases were identified on the basis of an ICD-9 diagnosis (487.xx) during the follow-up period. Influenza complications were defined as: a pneumonia diagnosis (ICD-9 codes 480.xx–486.xx) on or up to 30 days after an influenza diagnosis; diagnosis of stroke or myocardial infarction (MI; ICD-9 codes 410.xx, 430, 431, 432.x, 433.xx, 434.xx, 435.xx, 436) on or up to 30 days after an influenza diagnosis; or diagnosis of a neurological disorder (ICD-9 codes 320.xx–359.xx) on or up to 30 days after an influenza diagnosis.A single event (ICD-9 diagnosis), regardless of whether it was based on inpatient or outpatient records, was considered to be an outcome. The cohort entry date was that of the first of the two ICD9 diagnoses of RA; a set period of time between the two diagnoses was not specified.

Patients not continuously enrolled for 1 year before or after the date of cohort entry, and those with a diagnosis of RA or evidence of an RA-related disease in the year preceding the cohort entry, were excluded. A comparator group of patients who did not have RA was matched 1:1 according to age, gender and calendar year, with each case’s cohort entry date being matched to its control.

### Observations

Records of the following conditions or use of medication in the year prior to cohort entry were taken into account:

Conditions: cardiovascular disease, chronic lung disease, diabetes, cancer, HIV/AIDS, organ transplantation, neurological disorders and depression.

Disease-modifying anti-rheumatic drugs (DMARDs): auranofin, aurothioglucose, azathioprine, chloroquine, cyclophosphamide, cyclosporine, D-penicillamine, hydroxychloroquine, leflunomide, methotrexate, minocycline, sodium aurothiomalate and sulfasalazine.

Biologics: abatacept, adalimumab, anakinra, certolizumab, etanercept, golimumab, infliximab, rituximab and tocilizumab.

Influenza vaccination.

Patients were categorized into high or low corticosteroid use groups based on their average daily dose of corticosteroid use assessed in the 6-month period prior to cohort entry. High or low dose was defined as ≥10mg/day or <10mg/day the prednisone-equivalent dose, respectively. Oral glucocorticoid use for drugs other than prednisone was converted to prednisone-equivalent dosages (see Additional file 1 for conversion table]). The literature indicates that the use of high-dose corticosteroids can increase the risk of infection through immunosuppressive effects that induce cellular immunodeficiency, and that the lowest dosages possible are preferred [14]. Although data pertaining to low-dose corticosteroid use in patients with RA are as yet not conclusive [15], there does appear to be a link between high-dose corticosteroids and infectious complications (for example in patients with systemic lupus erythematosus [16]), and corticosteroid use has been associated with increased hospital mortality in patients with pandemic influenza A (H1N1) [17].

The observation period was measured in person-years from cohort entry until termination of enrolment in the health plan, date of the first diagnosis of influenza or a complication, or the end of the study period (whichever came first).

### Assessments

Baseline characteristics were assessed according to gender, age and presence of the conditions described earlier or use of the above medications or vaccines. Differences in the distributions for patients with an RA diagnosis versus controls were assessed by *χ*^2^ testing. Incidence rates of influenza and influenza-related complications were calculated as the number of new cases identified during the study period per 100,000 person-years for cases and controls. These rates were then stratified according to gender and age.

Incidence rate ratios (IRRs) for influenza and its complications, with 95% CIs, were calculated using Poisson regression (PROC GENMOD, SAS Institute Inc., Cary, NC, USA), with non-RA patients as reference, and stratification according to gender and age. Adjusted analyses accounted for differences in baseline characteristics. The statistical models adjusted for: ICD-9 claims for cardiovascular disease, chronic lung disease, transplantation and neurological disorders; and use of DMARDs, biologics, influenza vaccination, high-dose corticosteroids, low-dose corticosteroids, antibiotics and statins.

Analyses were repeated after exclusion of all patients with a history of DMARD or biological use in the 12 months preceding the date of RA diagnosis. Complications due to pneumonia or stroke/MI in patients with RA and controls were also examined, with age stratification (i) below 65 years and (ii) 65 years and over.

## Results

A total of 46,030 patients with RA and a matching number of controls were identified from the database (Table 1). Two-thirds of patients were female, and most patients were middle-aged or elderly. The largest single age group was 50–59 years (27.7% of patients), followed by 60–69 years and then ≥70 years. The median RA patient/control age was 57 years (Table 1). The most frequent comorbidities were cancer, cardiovascular disease and diabetes (Table 1). Cardiovascular disease was significantly more common in the RA group than in controls (18.10% vs 14.10%; p < 0.0001), but cancer and diabetes showed similar frequencies between the groups. Other significantly (p < 0.0001) more frequent conditions in patients with RA were chronic lung disease (5.37% vs 3.48%), transplantation (2.01% vs 0.91%) and neurological disorders (7.53% vs 0.4%).

**Table 1:** Baseline characteristics of patients identified from MarketScan

	**RA patients (cases)**		**Non-RA patients (controls)**		**p value**^**a**^
	**N**	**%**	**N**	**%**	
Total no. of patients	46,030	100	46,030	100	–
**Age (years)**					
Mean (SD)	56.61 (14.79)				–
Median (IQR)	57 (19)				–
18–29	1780	3.87	1780	3.87	1
30–39	4119	8.95	4119	8.95	1
40–49	8064	17.52	8064	17.52	1
50–59	12,735	27.67	12,735	27.67	1
60–69	10,428	22.65	10,428	22.65	1
≥70	8904	19.34	8904	19.34	1
**Medical conditions**					
Cardiovascular disease	8330	18.10	6489	14.10	<0.0001
Chronic lung disease	2470	5.37	1604	3.48	<0.0001
Diabetes	5284	11.48	5475	11.89	0.0501
Cancer	9152	19.88	9362	20.34	0.0842
HIV/AIDS	44	0.10	47	0.10	0.753
Transplant	926	2.01	421	0.91	<0.0001
Renal dysfunction	474	1.03	425	0.92	0.1005
Neurological disorders	3465	7.53	183	0.40	<0.0001
Depression	2555	5.55	2542	5.52	0.8514
**Medications at baseline**					
DMARDs	6413	13.93	642	1.39	<0.0001
Biologics	3276	7.12	104	0.23	<0.0001
Influenza vaccination	3500	7.60	4667	10.14	<0.0001
High-dose corticosteroids^b^	10,056	21.85	4990	10.84	<0.0001
Low-dose corticosteroids^b^	7903	17.17	1182	2.57	<0.0001
Antibiotics	28,847	62.67	24545	53.32	<0.0001
Statins	7408	16.09	7908	17.18	<0.0001
**RA patients who had received no active medication**					
No DMARDs, biologics or corticosteroids^c^	25,894	56.25	NA	NA	–
**Sex**					
Male	15,230	33.09	15230	33.09	1
Female	30,800	66.91	30800	66.91	1

Note: ^a^p value of 1 indicates cases with matched controls; ^b^Pharmacy data were used to compute an average daily dose during a 6-month period prior to cohort entry. The average daily dose was unique for each individual course of corticosteroid therapy. ‘High dose’ referred to values that were greater than or equal to the average daily dose, while ‘low dose’ referred to values that were less than the average daily dose; ^c^From 1 year prior to cohort entry until the end of the study; SD = standard deviation; IQR = interquartile range.

The two groups were significantly different (p < 0.0001) for all medications studied (Table 1). As expected, DMARDs, biological therapies and corticosteroids were significantly more frequently used by patients with RA than the controls; use of low-dose corticosteroids in particular was markedly more common in the RA group than the control group. Interestingly, influenza vaccination was more common in control patients than in those with RA (10.14% vs 7.60%). Statin and antibiotic consumption was significantly higher in the RA group, although the percentage difference was not as great as in other medication categories. Notably, of the 46,030 patients with RA, 20,136 (43.75%) had not received DMARDS, biological therapies or corticosteroids for their RA from 1 year prior to cohort entry up to the end of the study. Peak recruitment years were 2004 and 2007, with 42.51% of patients (39,134 of a total of 92,060 RA patients and controls) being drawn from the database in these years. Cohorts did not differ with respect to enrolment by calendar year over the entire period covered.

**Figure 1:**
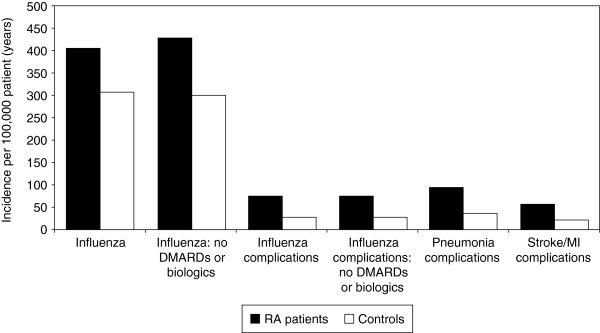
Incidence of influenza and complications in RA patients and controls recruited from the MarketScan database.

Overall, the incidence of influenza was higher in persons with RA than in controls (409.33 vs 306.12 cases per 100,000 patient-years; Table 2 and Figure 1). Incidence was higher in women than in men for both groups, although the increased incidence of influenza in RA cases over controls was particularly marked in men (61% increase vs 24% in women). The lowest incidence of influenza was in male controls; the highest was in female RA cases (Table 2). In terms of age, the highest incidence was seen in males aged 30–39 years. The lowest incidence was in controls aged 60–69 years (Table 2).

**Table 2:** Incidence rates (per 100,000 patient-years) of influenza and incidence rate ratios adjusted for baseline characteristics in patient subgroups

		**Incidence rates (per 100,000 patient-years) of influenza**						**Incidence rate ratios adjusted for baseline characteristics (95% CI)**		
**Parameter**	**Age categories**	**Males**		**Females**		**Total**		**Males**	**Females**	**Total**
		**RA cases**	**Controls**	**RA cases**	**Controls**	**RA cases**	**Controls**			
**Influenza**	18–29	495.81	336.70	534.54	444.42	522.13	409.48	1.72 (0.50, 5.94)	1.54 (0.70, 3.38)	1.57 (0.81, 3.03)
	30–39	761.26	347.99	633.31	387.97	670.90	375.64	2.07 (0.97, 4.38)	1.54 (0.93, 2.54)	1.65 (1.09, 2.51)
	40–49	521.10	379.23	515.93	498.87	517.57	459.64	1.06 (0.62, 1.82)	0.88 (0.62, 1.24)	0.92 (0.69, 1.24)
	50–59	358.50	245.10	381.06	357.66	373.58	319.45	1.38 (0.83, 2.32)	0.94 (0.68, 1.30)	1.06 (0.80, 1.38)
	60–69	299.32	106.51	366.56	200.68	342.11	165.94	2.44 (1.13, 5.26)	1.75 (1.11, 2.76)	1.94 (1.31, 2.86)
	≥70	309.20	238.81	314.97	252.78	312.93	247.95	0.97 (0.48, 1.96)	0.97 (0.58, 1.62)	0.98 (0.64, 1.48)
	**Overall**	**393.91**	**245.31**	**417.18**	**337.69**	**409.33**	**306.12**	**1.43 (1.09, 1.88)**	**1.13 (0.95, 1.35)**	**1.22 (1.05, 1.41)**
**Influenza complications**^**a**^	18–29	70.05	0	0	0	22.45	0	–	–	–
	30–39	57.49	0	71.98	0	67.71	0	–	–	–
	40–49	38.61	32.78	71.96	31.89	61.36	32.18	0.45 (0.06, 3.44)	1.45 (0.43, 4.93)	1.10 (0.39, 3.07)
	50–59	59.28	19.50	55.02	20.01	56.43	19.84	1.39 (0.26, 7.46)	2.06 (0.65, 6.47)	1.83 (0.71, 4.69)
	60–69	59.53	35.44	63.02	13.79	61.76	21.77	1.08 (0.25, 4.70)	3.17 (0.67, 14.97)	2.15 (0.77, 6.0)
	≥70	180.81	79.38	122.12	50.39	142.86	60.42	1.18 (0.39, 3.63)	1.76 (0.65, 4.74)	1.50 (0.71, 3.14)
	**Overall**	**80.28**	**34.08**	**71.95**	**23.55**	**74.76**	**27.15**	**1.30 (0.64, 2.63)**	**2.17 (1.22, 3.86)**	**1.82 (1.16, 2.81)**
**Influenza – no DMARDs or biologics**	18–29	541.94	343.41	609.33	456.87	587.11	419.88	1.72 (0.50, 5.49))	1.54 (0.71, 3.38)	1.57 (0.81, 3.04)
	30–39	804.93	352.17	670.75	377.68	710.76	369.80	2.07 (0.97, 4.38)	1.61 (0.97, 2.68)	1.71 (1.12, 2.61)
	40–49	516.81	383.82	526.27	490.36	523.19	455.34	1.08 (0.63, 1.85)	0.90 (0.63, 1.28)	0.94 (0.70, 1.27)
	50–59	387.17	247.13	399.08	358.47	395.08	320.48	1.38 (0.82, 2.31)	0.95 (0.69, 1.32)	1.06 (0.81, 1.40)
	60–69	354.97	107.65	413.55	203.48	391.99	168.05	2.42 (1.12, 5.25)	1.81 (1.14, 2.86)	1.97 (1.33, 2.91)
	≥70	304.73	240.80	270.23	238.58	282.70	239.35	0.93 (0.45, 1.92)	0.99 (0.57, 1.70)	0.96 (0.62, 1.49)
	**Overall**	**426.42**	**247.82**	**437.87**	**333.77**	**433.96**	**304.30**	**1.44 (1.09, 1.89)**	**1.17 (0.98, 1.40)**	**1.25 (1.07, 1.45)**
	18–29	76.49	0	0	0	25.21	0	0	–	–
	30–39	60.73	0	77.64	0	72.59	0	0	–	–
	40–49	41.40	33.17	79.96	32.39	67.40	32.64	0.46 (0.06, 3.44)	1.45 (0.43, 4.93)	1.10 (0.39, 3.07)
	50–59	65.34	19.66	57.68	20.35	60.25	20.11	1.39 (0.26, 7.46)	1.98 (0.62, 6.27)	1.77 (0.69, 4.56)
	60–69	68.26	35.82	72.71	13.98	71.07	22.04	1.02 (0.23, 4.55)	3.56 (0.76, 16.66)	2.13 (0.76, 6.01)
	≥70	145.29	80.04	112.16	42.47	124.13	55.49	0.99 (0.30, 3.28)	1.83 (0.62, 5.35)	1.40 (0.63, 3.08)
	**Overall**	**76.64**	**34.43**	**74.20**	**22.41**	**75.03**	**26.53**	**1.23 (0.59, 2.51)**	**2.23 (1.23, 4.03)**	**1.76 (1.12, 2.77)**
**Pneumonia complications**^**b**^	<65	49.38	31.92	90.34	34.93	76.75	33.91	1.00 (0.40, 2.45)	2.20 (1.26, 3.85)	1.78 (1.11, 2.85)
	≥65	134.89	29.83	141.59	47.02	139.25	41.10	1.66 (0.44, 6.26)	2.12 (0.96, 4.68)	2.01 (1.02, 3.95)
	**Overall**	**76.22**	**31.33**	**105.54**	**38.34**	**95.66**	**35.95**	**1.15 (0.55, 2.41)**	**2.15 (1.36, 3.38)**	**1.84 (1.25, 2.70)**
**Stroke/MI complications**^**c**^	<65	52.46	11.97	32.09	18.48	38.83	16.27	3.03 (0.85, 10.83)	1.27 (0.56, 2.90)	1.72 (0.87, 3.40)
	≥65	87.68	19.90	101.67	47.03	96.78	37.68	2.11 (0.42, 10.50)	1.55 (0.68, 3.50)	1.68 (0.82, 3.47)
	**Overall**	**63.52**	**14.24**	**52.70**	**26.53**	**56.34**	**22.34**	**2.69 (1.0, 7.28)**	**1.37 (0.77, 2.44)**	**1.69 (1.02, 2.75)**

Note: ^a^Influenza complications = pneumonia diagnosis (ICD-9 codes 480.xx–486.xx); stroke or MI (ICD-9 codes 410.xx, 430, 431, 432.x, 433.xx, 434.xx, 435.xx, 436); or neurological disorder (ICD-9 codes 320.xx–359.xx); all on or up to 30 days after an influenza diagnosis; ^b^ICD-9 codes 480.xx–486.xx; ^c^ICD-9 codes 410.xx, 430, 431, 432.x, 433.xx, 434.xx, 435.xx, 436.

The incidence of influenza complications was much higher in patients with RA than in controls (2.75-fold increase overall; Table 2 and Figure 1). Complications were most frequent in patients aged ≥70 years: frequencies in RA patients were more than twice those in controls, with the highest incidence of all in men with RA aged ≥70 years (Table 2).

Presence or absence of DMARDs or biological therapy made little or no difference overall to the incidence of influenza or its complications in either patients with RA or controls (Table 2 and Figure 1), although there was a shifting of influenza incidence across age groups when compared with the overall results. The incidence of influenza among patients with RA was markedly increased in the absence of DMARDs or biologics, with clustering in the younger age groups (18–49 years). The peak incidence of 804.93/100,000 patient-years was in men with RA aged 30–39 years (Table 2).

Frequencies of pneumonia and stroke/MI complications were both higher (to a similar extent overall to influenza complications generally; Figure 1) in patients with RA. As might be expected, rates were higher in patients aged 65 years and over for both RA and control groups, with the highest frequencies observed in women in this age group with RA (Table 2).

**Figure 2:**
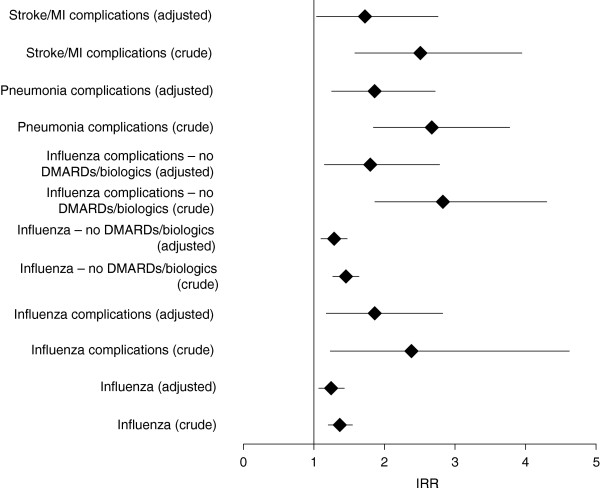
**Forest plot showing IRRs for influenza and complications in patients drawn from the MarketScan database.** IRRs (RA patients versus controls) are shown for each endpoint as overall figures for patients of all ages. Adjusted IRRs are those with baseline factors accounted for.

Crude IRRs (Figure 2) and those adjusted for baseline characteristics (Table 2 and Figure 2) showed significantly increased rates of influenza or influenza complications in the overall population when RA was present, although this effect was not observed in some patient subgroups (see below). In the overall population (all ages), the greatest increases in risk before baseline adjustment were noted for influenza complications (all patients and those not taking DMARDs or biologics), pneumonia complications, and stroke/MI complications (Figure 2). The crude pneumonia complication risk was more than doubled in patients with RA (Figure 2).

The risk of influenza itself was increased significantly in patients (combined genders) aged 60–69 years, with most of this risk increase being accounted for by men (Table 2). There was also a noteworthy increase in risk in men aged 30–39 years. Similar results were observed in patients with no history of use of DMARDs or biological therapies (Table 2).

Note that in some age groups (18–29 and 30–39 years), IRRs for influenza complications could not be calculated. This was because the control groups in these strata had no record of influenza complications. Most other age groups showed an increase in risk of complications in patients with RA without attaining statistical significance after adjustment for baseline factors, although significance was reached in women and both genders combined (but not in men alone) when data for all ages were combined (Table 2). Men aged 40–49 years with RA showed a non-significant reduction in risk of influenza complications (IRR = 0.45; 95% CI: 0.06, 3.44).

In men with RA with no history of DMARD or biological use, there was an approximate halving of the risk of influenza complications in men aged 40–49 years (IRR = 0.46; 95% CI: 0.06, 3.44), and an increase in men aged 50–59 years (IRR = 1.39; 95% CI: 0.26, 7.46; Table 2). In general, however, the risk of influenza complications was similar in RA patients not receiving DMARDs or biologics to that seen in all RA patients (Table 2).

Adjusted IRRs (Table 2) showed an increased rate of pneumonia among women with RA, with the greatest risk observed among women aged <65 years. Stroke/MI complications were increased to a greater extent in men both before and after baseline adjustment, although statistical significance was borderline after adjustment (Table 2 and Figure 2).

## Discussion

In this sample of patients from the MarketScan database, the incidence of influenza was higher in those with RA than in matched controls. After adjustment for baseline factors, the risk of influenza was significantly increased in patients with RA, with both genders aged 60–69 years and men aged 30–39 years being particularly at risk. Of note is the observation that the incidence of influenza was increased similarly in patients not taking DMARDs or biological therapies and in the overall RA population.

Risks of complications could not be calculated or were not significant in some individual age groups and genders, although pooled data from all ages generally showed increased IRRs after adjustment for baseline factors, regardless of whether or not DMARDs or biologics were being taken. When individual age groups were analysed, women with RA appeared to be at greater risk of influenza complications than men. Analysis of individual age groups showed the risk of pneumonia to be increased significantly in women aged <65 years (in contrast to the results before baseline corrections were applied); stroke/MI risk was increased to a greater extent in men with RA.

Infections noted as complications of RA have been reported frequently over the past few decades. In particular, septic arthritis and pulmonary infections have been described since the 1960s/70s [18,19]. Studies in smaller cohorts of patients have shown results similar to those in the present study for rates of infection generally. Doran et al. [1] found that patients with RA have nearly twice the rate of infection seen in controls (although these authors excluded patients with upper respiratory tract infections). This study included 609 patients with RA and 609 matched controls, with median age 58 years, and showed hazard ratios of 1.70, 1.83 and 1.45 for objectively confirmed infections, infections requiring hospitalisation and any documented infection, respectively, in patients with RA. The respiratory tract was identified as an infection site of high risk.

Potential explanations for increased risk of infection generally in patients with RA include immunological abnormalities involving circulating T cells, which may impair the ability of the immune system to respond to infection. One study has shown a marked reduction in the ability of patients with RA to produce T cells and to maintain T cell homeostasis [20]. Impaired thymic function may also be involved, with increased T cell turnover in the periphery being a secondary mechanism [20]. Other factors may include those related to RA itself (poor mobility or effects of joint surgery) or its extra-articular manifestations. Doran et al. [1] also cited comorbidities such as diabetes as potential contributing factors to increased risk of infection in RA, but in the present study risk of influenza specifically persisted after commonly observed chronic comorbidities were accounted for by IRR adjustment.

The use of DMARDs and biologics is reported to increase risk of infection generally in RA patients [21,22]. Corticosteroids have many immunosuppressive effects that induce cellular immunodeficiency, necessitating careful timing of administration to accompany diurnal cortisol secretion patterns and the use of the lowest possible dosages [21]. Moreover, tumour necrosis factor (TNF)-α plays a key role in the pathogenesis of RA, and the propensity of anti-TNF-α agents to facilitate infection in patients with RA is a source of concern. This is reflected in the recent labelling update issued by the Food and Drug Administration [23], in which the warnings and precautions for TNF-α inhibitors have been revised to ensure consistency of information on the risk of serious infections and the associated pathogens. Most notably, the ‘Boxed Warning’ for these agents now includes the risk of infection from Legionella and Listeria. Patients and physicians are urged to be vigilant for signs of infection when using these drugs [21].

Despite the above concerns, Furst [22] has reported that influenza vaccination is effective in the presence of TNF inhibitors or abatacept. We found similar increases in risk whether DMARDs/biologics were used or not after controlling for baseline factors including vaccination. We note however, that the present study, unlike those cited above, focused exclusively on influenza in patients with RA rather than on infectious disease in general. Most recently, data obtained in 340 patients with RA and 234 control patients at a Brazilian hospital have shown seroprotection rates after immunisation to be more than 20% lower for RA patients than for patients who did not have the disease (60.1% vs 82.9%; p < 0.001) [24]. Seroconversion rates were similarly reduced (antibodies in 53.4% of RA patients and 76.9% of controls; p < 0.001).

The Centers for Disease Control and Prevention (CDC) state that persons with RA have an increased risk of respiratory infections such as influenza, together with a raised risk of complications and hospitalisation [25]. In their guidance for the 2010–2011 season, the CDC recommended annual vaccination for all persons aged 6 months or over, with protection of persons at higher risk for influenza complications continuing to be a focus for vaccination efforts [26]. Although not specifically mentioned in these guidelines, patients with inflammatory forms of arthritis (including RA) are now listed as an at-risk group in a September 2010 update on arthritis and influenza [25].

The increased risk of pneumonia in this study is in accordance with UK data based on a large primary care database that showed an adjusted OR for pneumonia of 1.84 in patients of all ages with RA (n = 387) relative to controls (n = 821) [9]. These researchers found, as in the present study, that adjustment for potentially confounding baseline variables tended to reduce risk.

Although we showed increased risk of influenza in patients with RA regardless of whether DMARDs or biologics were used or not, our study was limited by its inability to show the effect, if any, of the use of these agents on the severity of influenza infection. Such an effect requires further investigation in light of the continuing progress being made with drugs that target the immunological mechanisms underlying RA. In addition, it is not clear whether the results of this study sample are generalisable to the overall US population. Inclusion/exclusion criteria were not especially strict. Patients not continuously enrolled for 1 year before or after cohort entry, and those with a diagnosis of RA or evidence of an RA-related disease in the year preceding the cohort entry, were excluded. While RA patients are likely to be immunocompromised, it is unlikely that a health professional would closely monitor an RA patient specifically for influenza or influenza complications compared with anyone else in the general population. Thus, any selection bias may be non-differential and minimal. In addition, the statistical models accounted for baseline differences in the RA and matched comparison cohorts. Similarly, any misclassification of an influenza-associated complication would be no more likely to occur in an RA cohort than in the comparison group. Nevertheless, this study may be among the few to examine the association between RA and influenza or its complications in such a large patient population with or without prescription of DMARDs or biologics.

## Conclusions

RA is known to be a risk factor for increased incidence and risk of complications of infectious diseases. Previously published data have shown this increase in risk to include upper respiratory disease, notably pneumonia. Analysis of gender- and age-specific subgroups showed in particular that women were at greater risk than men for complications generally, but that risk of stroke and MI complications was increased mainly in men. The data presented here show that RA is associated with increased risk of seasonal influenza and its complications specifically, concordant with the position now being taken by the CDC on influenza risk in patients with inflammatory rheumatic disease; the prescription of DMARDs or biologics appeared not to affect the incidence of influenza or its complications in RA patients.

## Competing interests

All authors are employees of (WAB, AA, PN) or consultants to (ST) Roche or Genentech (a member of the Roche Group).

## Authors’ contributions

All authors contributed to the design, conduct, analysis and/or interpretation of the investigation reported herein. All authors participated in the preparation, review and approval of this article.

## Pre-publication history

The pre-publication history for this paper can be accessed here:

http://www.biomedcentral.com/1471-2474/13/158/prepub

## Supplementary Material

Additional file 1 **Conversion table for prednisone-equivalent dosages.** (DOCX 11 kb) Click here for file

## References

[B1] Doran MF Crowson CS Pond GR O'Fallon WM Gabriel SE Frequency of infection in patients with rheumatoid arthritis compared with controls: a population-based study Arthritis Rheum 2002 46 2287 2293 10.1002/art.10524 12355475

[B2] Glück T Müller-Ladner U Vaccination in patients with chronic rheumatic or autoimmune diseases Clin Infect Dis 2008 46 1459 1465 10.1086/587063 18419456

[B3] Baum J Infection in rheumatoid arthritis Arthritis Rheum 1971 14 135 137 10.1002/art.1780140119 5542366

[B4] Goldenberg DL Infectious arthritis complicating rheumatoid arthritis and other chronic rheumatic disorders Arthritis Rheum 1989 32 496 502 10.1002/anr.1780320422 2650687

[B5] Housden MM Bell G Heycock CR Hamilton J Saravanan V Kelly CA How to reduce morbidity and mortality from chest infections in rheumatoid arthritis Clin Med 2010 10 326 329 20849003 10.7861/clinmedicine.10-4-326 PMC4952158

[B6] Gershon S Wise RP Niu M Siegel J Postlicensure reports of infection during use of etanercept and infliximab [abstract] Arthritis Rheum 2000 43 2857

[B7] Baghai M Osmon DR Wolk DM Wold LE Haidukewych GJ Matteson EL Fatal sepsis in a patient with rheumatoid arthritis treated with etanercept Mayo Clin Proc 2001 76 653 656 11393506 10.4065/76.6.653

[B8] Lee JH Slifman NR Gershon SK Edwards ET Schwieterman WD Siegel JN Wise RP Brown SL Udall JN Jr Braun MM Life-threatening histoplasmosis complicating immunotherapy with tumor necrosis factor alpha antagonists infliximab and etanercept Arthritis Rheum 2002 46 2565 2570 10.1002/art.10583 12384912

[B9] Vinogradova Y Hippisley-Cox J Coupland C Identification of new risk factors for pneumonia: population-based case-control study Br J Gen Pract 2009 59 e329 e338 19843413 10.3399/bjgp09X472629 PMC2751937

[B10] Department of Health PL CMO (2005)1: the Pneumococcal Immunisation Programme for Older People and Risk Groups 2009 London: Department of Health http://www.dh.gov.uk/en/Publicationsandstatistics/Lettersandcirculars/Professionalletters/Chiefmedicalofficerletters/DH_4107903

[B11] Mayo Clinic Influenza (flu). Risk factors. 21 August 2010 http://www.mayoclinic.com/health/influenza/DS00081/DSECTION=risk-factors

[B12] Thomson Reuters Pharmaceuticals: Data, Databases and Online Tools: Marketscan Research Databases 2011 New York, NY, USA: Thomson Reuters http://thomsonreuters.com/products_services/healthcare/healthcare_products/pharmaceuticals/mktscan_res_db/

[B13] Centers for Disease Control and Prevention Classification of diseases, functioning, and disability. International Classification of Diseases, Ninth Revision (ICD-9) 2009 Atlanta, GA, USA: CDC http://www.cdc.gov/nchs/icd/icd9.htm

[B14] Cutolo M Seriolo B Pizzorni C Secchi ME Soldano S Paolino S Montagna P Sulli A Use of glucocorticoids and risk of infections Autoimmun Rev 2008 8 153 155 10.1016/j.autrev.2008.07.010 18703175

[B15] Ruyssen-Witrand A Fautrel B Saraux A Le-Loët X Pham T Infections induced by low-dose corticosteroids in rheumatoid arthritis: a systematic literature review Joint Bone Spine 2010 77 246 251 10.1016/j.jbspin.2010.02.009 20451437

[B16] Kang I Park SH Infectious complications in SLE after immunosuppressive therapies Curr Opin Rheumatol 2003 15 528 534 10.1097/00002281-200309000-00002 12960476

[B17] Xi X Xu Y Jiang L Li A Duan J Du B Chinese Critical Care Clinical Trial Group: Hospitalized adult patients with 2009 influenza A(H1N1) in Beijing China: risk factors for hospital mortality BMC Infect Dis 2010 10 256 20799934 10.1186/1471-2334-10-256 PMC2941683

[B18] Mitchell WS Brooks PM Stevenson RD Buchanan WW Septic arthritis in patients with rheumatoid disease: a still underdiagnosed complication J Rheumatol 1976 3 124 133 950628

[B19] Rimoin DL Wennberg JE Acute septic arthritis complicating chronic rheumatoid arthritis JAMA 1966 196 617 621 10.1001/jama.1966.03100200057018 5952288

[B20] Koetz K Bryl E Spickschen K O'Fallon WM Goronzy JJ Weyand CM T cell homeostasis in patients with rheumatoid arthritis Proc Natl Acad Sci U S A 2000 97 9203 9208 10.1073/pnas.97.16.9203 10922071 PMC16846

[B21] Atzeni F Bendtzen K Bobbio-Pallavicini F Conti F Cutolo M Montecucco C Sulli A Valesini G Sarzi-Puttini P Infections and treatment of patients with rheumatic diseases Clin Exp Rheumatol 2008 26 Suppl 48 S67 S73 18570757

[B22] Furst DE The risk of infections with biologic therapies for rheumatoid arthritis Semin Arthritis Rheum 2010 39 327 346 10.1016/j.semarthrit.2008.10.002 19117595

[B23] FDA US Food and Drug Administration: Tumor Necrosis Factor-alpha (TNFα) Blockers: Label Change - Boxed Warning Updated for Risk of Infection from Legionella and Listeria including Remicade (infliximab), Enbrel (etanercept), Humira (adalimumab), Cimzia (certolizumab pegol), and Simponi (golimumab). FDA 2011 http://www.fda.gov/Safety/MedWatch/SafetyInformation/SafetyAlertsforHumanMedicalProducts/ucm270977.htm

[B24] Ribeiro A Guedes L Moraes J Saad C Aikawa N Calish A França I Gonçalves C Sampaio-Barros P Carvalho J Borba E Timenetsky M Precioso A Duarte A Bonfa E Laurindo I Implications of reduced seroprotection (SP) after pandemic H1N1 (2009) influenza adjuvant-free vaccination in patients with rheumatoid arthritis on active systemic treatment (classical and biologic DMARDS) Ann Rheum Dis 2011 70 Suppl 3 117 21859696 10.1136/ard.2011.152983

[B25] Centers for Disease Control and Prevention Arthritis and Influenza Update 2010-2011 Atlanta, GA, USA: CDC 14 September 2010. http://www.cdc.gov/arthritis/flu.htm

[B26] Fiore AE Uyeki TM Broder K Finelli L Euler GL Singleton JA Iskander JK Wortley PM Shay DK Bresee JS Cox NJ Centers for Disease Control and Prevention (CDC) Centers for Disease Control and Prevention (CDC) Prevention and control of influenza with vaccines: recommendations of the Advisory Committee on Immunization Practices (ACIP), 2010 MMWR Recomm Rep 2010 59 1 62 20689501

