# Supplementary material for: Rheumatoid arthritis and the incidence of influenza and influenza-related complications: a retrospective cohort study
Source: BMC Musculoskelet Disord. 2012 Aug 27;13:158. doi: 10.1186/1471-2474-13-158 (PMC3495205; doi:10.1186/1471-2474-13-158)
Supplement: Additional file 1 — Conversion table for prednisone-equivalent dosages. (DOCX 11 kb) [file 1471-2474-13-158-S1.docx]

**Conversion table for prednisone-equivalent dosages**

|  | **High-dose equivalents, average daily dose ≥ (mg)** | **Low-dose equivalents, average daily dose < (mg)** |
| --- | --- | --- |
| **Prednisone** | 10 | 10 |
| **Betamethasone** | 1.5 | 1.5 |
| **Cortisone** | 50 | 50 |
| **Dexamethasone** | 1.5 | 1.5 |
| **Hydrocortisone** | 40 | 40 |
| **Methylprednisone** | 8 | 8 |
| **Prednisolone** | 10 | 10 |
| **Triamcinolone** | 8 | 8 |
